# Supplementary material for: Chromosome-Length Assembly of the Baikal Seal (Pusa sibirica) Genome Reveals a Historically Large Population Prior to Isolation in Lake Baikal
Source: Genes (Basel). 2023 Feb 28;14(3):619. doi: 10.3390/genes14030619 (PMC10048373; doi:10.3390/genes14030619)
Supplement: Supplementary file 1 [file genes-14-00619-s001.zip › genes-2143573-supplementary.pdf]

Table S1: Description of baikal seal libraries;

Table S2. Read counts of *Pusa sibirica* and additional pinniped samples before and after trimming;

Table S3. Genome completeness metrics based on orthologs analysis with BUSCO ver.5 using carnivora database (carnivora_odb10);

Table S4. Major types of transposable elements detected in the genome of baikal seal;

Table S5. Chromosome assignments;

Table S6. Statistical metrics of heterozygosity for analyzed samples of three pinniped species excluding X chromosome. SNPs are counted in 1 Mbp sliding windows with 100kbp step, and scaled to SNPs per kbp;

Table S7. Table with calibrations used for dating in phylogeny analysis;

Table S8. Dating of divergence times based on dated maximum likelihood phylogenetic tree of 5 seals (*Erignathus barbatus*, *Phoca vitulina*, *Phoca largha*, *Halichoerus grypus*, *Pusa sibirica*) and 6 other carnivore species (*Acinonyx jubatus*, *Puma concolor*, *Canis lupus familiaris*, *Ursus arctos*, *Ailurus fulgens*, *Odobenus rosmarus*) using 5 fossil calibrations;

Figure S1. K-mer distribution of 23-mers for *Pusa sibirica, Halichoerus grypus,* and *Phoca largha* read after standart adapters trimming (A), and after further cut length, and downsampling (B). PUSI_M - *Pusa sibirica* male sample; PUSI_F - *Pusa sibirica* female sample; HAGR_M - *Halichoerus grypus* DNAZoo sample; PHLA_F_DZ - *Phoca largha* DNAZoo sample; PHLA_F_SU - *Phoca largha* Seoul University sample;

Figure S2. Coverage plots for (A) *Pusa sibirica,* female, DNAZoo, (B) *Pusa sibirica,* male, DNAZoo, (C) *Halichoerus grypus,* male, DNAZoo, (D) *Phoca largha,* female, DNAZoo, and (E) *Phoca largha,* female, Seoul University. Coverage was calculated in non-overlapping sliding windows of 1 Mbp and divided by whole genome median coverage;

Figure S3. Genome-wide heterozygosity distribution for 2 samples of baikal seal (*Pusa sibirica*), 1 sample of grey seal (*Halichoerus grypus*) and 2 samples of spotted seal (*Phoca largha*). SNPs are counted in 1 Mbp non-overlapping sliding windows and scaled to heterozygous SNPs per kbp;

Figure S4. Demographic history reconstruction for all available samples of *Pusa sibirica, Halichoerus grypus*, and *Phoca largha* with mutation rate $2.5*10^{-8}$ excluding X chromosome;

Figure S5. The phylogenetic tree for eleven carnivores including five seal species (*Halichoerus grypus*, *Pusa sibirica, Phoca largha*, *Phoca vitulina* and *Erignathus barbatus*). Node labels show posterior probabilities. Branch lengths are in proportion to expected changes per site.

Table S1. Description of baikal seal libraries.

Library ID	SRA ID	Library type	Sequencer	Insert size (target), bp	Insert size (actual), bp	Read length, bp	Reads, million	
							raw	filtered
male	SRR22409084	PE	HiSeq2500	350	300	100	2386.3	2341.8
mp3kb	SRR22409081	MP	HiSeq2500	3000	2800	100	593.4	210.1
gelfree	SRR22409083	MP	HiSeq2500	3000	3200	100	33.8	13.3
MP3-4	SRR22409082	MP	HiSeq2500	3000-4000	3200	100	34.8	14.5
MP5-7	SRR22409080	MP	HiSeq2500	5000-7000	5100	100	31.2	13.3
MP8-10	SRR22409079	MP	HiSeq2500	8000-10000	8400	100	41.9	17.6
HiC		HiC	NovaSeq6000	NA	NA	150	1348.5	1013.9

Table S2. Read counts of *Pusa sibirica* and additional pinniped samples before and after trimming.

Species	Raw read pairs, mln	Trimmed, mln	Downsampled, mln	Saved reads, %
Pusa sibirica (male, DNAZoo); SRR22409084	1 193.1	1129.2	338.9	28.4
Pusa sibirica (female, DNAZoo); SRR22409450	357.4	340.6	340.6	95.3
Halichoerus grypus (male, DNAZoo); SRR16086823	458.3	434.1	338.6	73.4
Phoca largha (female, DNAZoo); SRR13167966	355.4	337.8	337.8	95
Phoca largha (female, Seoul National University); SRR6433059	504.5	452.4	361.9	71.7

Table S3. Genome completeness metrics based on orthologs analysis with BUSCO ver.5 using carnivora database (carnivora_odb10).

Species	Complete single-copy	Complete duplicated	Fragmented	Missing
Erignathus barbatus	12931 (89.2%)	293 (2.0%)	274 (1.9%)	1004 (6.9%)
Halichoerus grypus	12955 (89.3%)	302 (2.1%)	270 (1.9%)	975 (6.7%)
Odobenus rosmarus	13353 (92.1%)	473 (3.3%)	126 (0.9%)	550 (3.7%)
Phoca largha	13053 (90.0%)	291 (2.0%)	235 (1.6%)	923 (6.4%)
Phoca vitulina	13412 (92.5%)	352 (2.4%)	142 (1.0%)	596 (4.1%)
Pusa sibirica	12755 (88.0%)	225 (1.6%)	406 (2.8%)	1116 (7.6%)

Table S4. Major types of transposable elements detected in the genome of baikal seal.

Type of element	*Halichoerus grypus*		*Phoca largha*		*Pusa sibirica*		*Phoca vitulina*	
	**%**	**Mbp**	**%**	**Mbp**	**%**	**Mbp**	**%**	**Mbp**
SINEs	3.14	76	3.15	74	3.16	75	3.16	75
LINEs	19.25	465	18.5	434	19.18	453	20.67	489
LTR elements	4.92	119	4.85	114	4.92	116	4.84	114
DNA elements	3.15	76	3.12	73	3.16	75	3.12	74
Unclassified	0.03	0.6	0.03	0.6	0.03	0.6	0.03	0.6
Total	30.49	736	29.64	696	30.46	719	31.81	752

Table S5. Chromosome assignments.

Pusa sibirica		*Halichoerus grypus*		*Phoca largha*		*Phoca vitulina*	
**Scaffold**[1]	**Chr**[2]	**Scaffold**	**Chr**	**Scaffold**	**Chr**	**Scaffold**	**Chr**
HiC_scaffold_2	Chr1	HiC_scaffold_2	Aut2	HiC_scaffold_2	Aut2	HiC_scaffold_2	Chr1
HiC_scaffold_1	Chr2	HiC_scaffold_1	Aut1	HiC_scaffold_1	Aut1	HiC_scaffold_4	Chr2
HiC_scaffold_3	Chr3	HiC_scaffold_3	Aut3	HiC_scaffold_3	Aut3	HiC_scaffold_16	Chr3
HiC_scaffold_4	Chr4	HiC_scaffold_4	Aut4	HiC_scaffold_4	Aut4	HiC_scaffold_1	Chr4
HiC_scaffold_5	Chr5	HiC_scaffold_5	Aut5	HiC_scaffold_5	Aut5	HiC_scaffold_3	Chr5
HiC_scaffold_6	Chr6	HiC_scaffold_6	Aut6	HiC_scaffold_6	Aut6	HiC_scaffold_12	Chr6
HiC_scaffold_7	Chr7	HiC_scaffold_7	Aut7	HiC_scaffold_7	Aut7	HiC_scaffold_13	Chr7
HiC_scaffold_8	Chr8	HiC_scaffold_8	Aut8	HiC_scaffold_8	Aut8	HiC_scaffold_14	Chr8
HiC_scaffold_9	Chr9	HiC_scaffold_9	Aut9	HiC_scaffold_9	Aut9	HiC_scaffold_11	Chr9
HiC_scaffold_10	Chr10	HiC_scaffold_10	Aut10	HiC_scaffold_10	Aut10	HiC_scaffold_6	Chr10
HiC_scaffold_11	Chr11	HiC_scaffold_11	Aut11	HiC_scaffold_11	Aut11	HiC_scaffold_10	Chr11
HiC_scaffold_12	Chr12	HiC_scaffold_12	Aut12	HiC_scaffold_12	Aut12	HiC_scaffold_5	Chr12
HiC_scaffold_14	Chr13	HiC_scaffold_14	Aut14	HiC_scaffold_14	Aut14	HiC_scaffold_9	Chr13
HiC_scaffold_13	Chr14	HiC_scaffold_13	Aut13	HiC_scaffold_13	Aut13	HiC_scaffold_7	Chr14
HiC_scaffold_15	Chr15	HiC_scaffold_15	Aut15	HiC_scaffold_15	Aut15	HiC_scaffold_8	Chr15
HiC_scaffold_16	ChrX	HiC_scaffold_16	ChrX	HiC_scaffold_16	ChrX	HiC_scaffold_15	ChrX

[1] Scaffold name according to assembly;

[2] Chromosome name mapped with scaffold according to fluorescent *in situ* hybridization (FISH).

Table S6. Statistical metrics of heterozygosity for analyzed samples of three pinniped species excluding X chromosome. SNPs are counted in 1 Mbp sliding windows with 100kbp step, and scaled to SNPs per kbp.

Species	min	median	mean	mode	max
Halichoerus grypus, male	0	0.5	0.51	0.48 [1]	1.72
Phoca largha, female (DNAZoo)	0	0.68	0.7	0.67	3.23
Phoca largha, female (Seoul university)	0	0.63	0.64	0.61	2
Pusa sibirica, male	0	0.62	0.66	0.56[*]	2
Pusa sibirica, female	0	0.67	0.7	0.56	2.34

[1] for male samples we observed additional mode at 0. It corresponds to hemizigous region (part of X chromosome outside of PAR) in males.

Table S7. Table with calibrations used for dating in phylogeny analysis.

MRCA [1]	Fossil calibrations		Source
	Lower boundary	Upper boundary	
Feliformia - Caniformia	37.3	66	Benton et al. 2015; Fossil Calibration Database [2]
Musteloidea - Pinnipedia	33.8	48.8	Meredith et al. 2011
Phocidae - Otarioidea	20.4	34	Meredith et al. 2011
Erignathus - Pusa+Phoca	-	23	Hassanin et al, 2021
Phoca - Pusa	0.79	-	Fulton and Stobeck, 2010; Demere et al., 2003

[1] Most recent common ancestor.

[2] fossilcalibrations.org

Table S8. Dating of divergence times based on dated maximum likelihood phylogenetic tree of 5 seals (*Erignathus barbatus, Phoca vitulina, Phoca largha, Halichoerus grypus, Pusa sibirica*) and 6 other carnivore species (*Acinonyx jubatus, Puma concolor, Canis lupus familiaris, Ursus arctos, Ailurus fulgens, Odobenus rosmarus*) using 5 fossil calibrations.

MRCA [1]	Independent clock, million years			Correlated clock, million years			Global clock, million years		
	Node age	95% CI LB [2]	95% CI UB [3]	Node age	95% CI LB	95% CI UB	Node age	95% CI LB	95% CI UB
Feliformia - Caniformia	62.8	54	67.6	59.6	48.6	66.5	66.8	63	72
Canidae - Ursidae	57.6	47.4	65.4	56.9	46.6	64.5	57.7	54.4	62.2
Ursidae - Ailuridae	44.7	36.9	51.6	45.9	38	51.6	42.2	39.7	45.4
Musteloidea - Pinnipedia	41.4	34.2	47.9	43.3	35.9	48.7	39.6	37.4	42.7
Phocidae - Odobenidae	21.5	19.5	25.5	24.3	20.3	30.2	16.2	15.3	17.5
Erignathus - Pusa+Phoca (underlined)	7.1	4.8	9.6	11.2	8.3	15.4	6.3	5.9	6.8
Phoca - Pusa+Halicoerus grypus (underlined)	2	1.4	2.6	3.4	2.4	5.1	1.8	1.7	2
Pusa sibirica - Halicoerus grypus	1.6	1.1	2.2	2.9	2	4.3	1.6	1.5	1.7
Phoca largha - Phoca vitulina	0.7	0.4	1	1.3	0.9	2	0.6	0.6	0.7

[1] Most recent common ancestor

[2] Lower boundary of 95 % confidence interval for Node age

[3] Upper boundary of 95 % confidence interval for Node age

For bodes in **bold** both lower and upper boundaries based on fossil evidence was set, for nodes in underlined - only one, lower or upper, respectively.

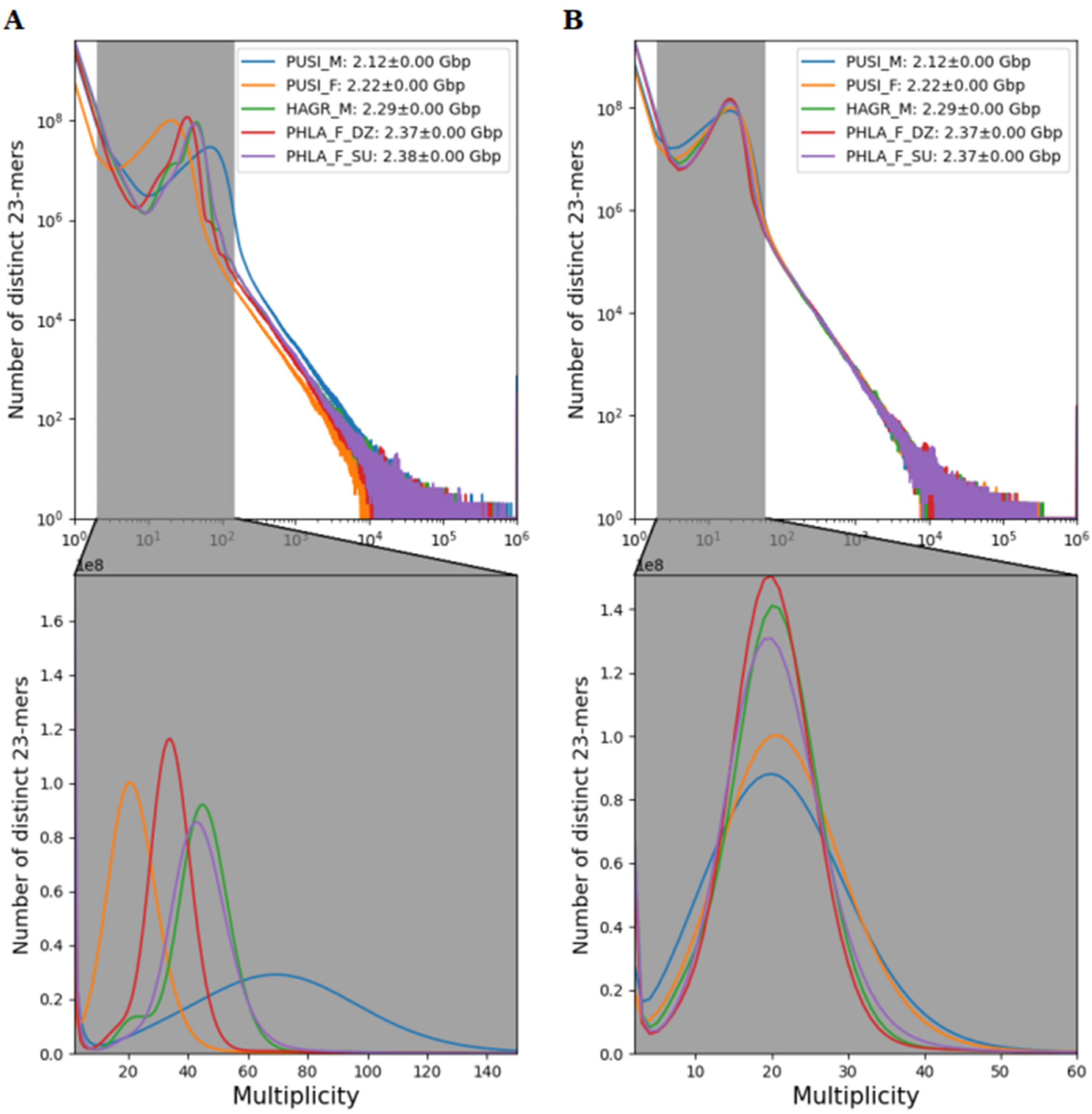

Figure S1. K-mer **d**istribution of 23-mers for *Pusa sibirica, Halichoerus grypus,* and *Phoca largha* read after standart adapters trimming (**A**), and after further cut length, and downsampling (**B**). **PUSI_M** - *Pusa sibirica* male sample; **PUSI_F** - *Pusa sibirica* female sample; **HAGR_M** - *Halichoerus grypus* DNAZoo sample; **PHLA_F_DZ** - *Phoca largha* DNAZoo sample; **PHLA_F_SU** - *Phoca largha* Seoul University sample.

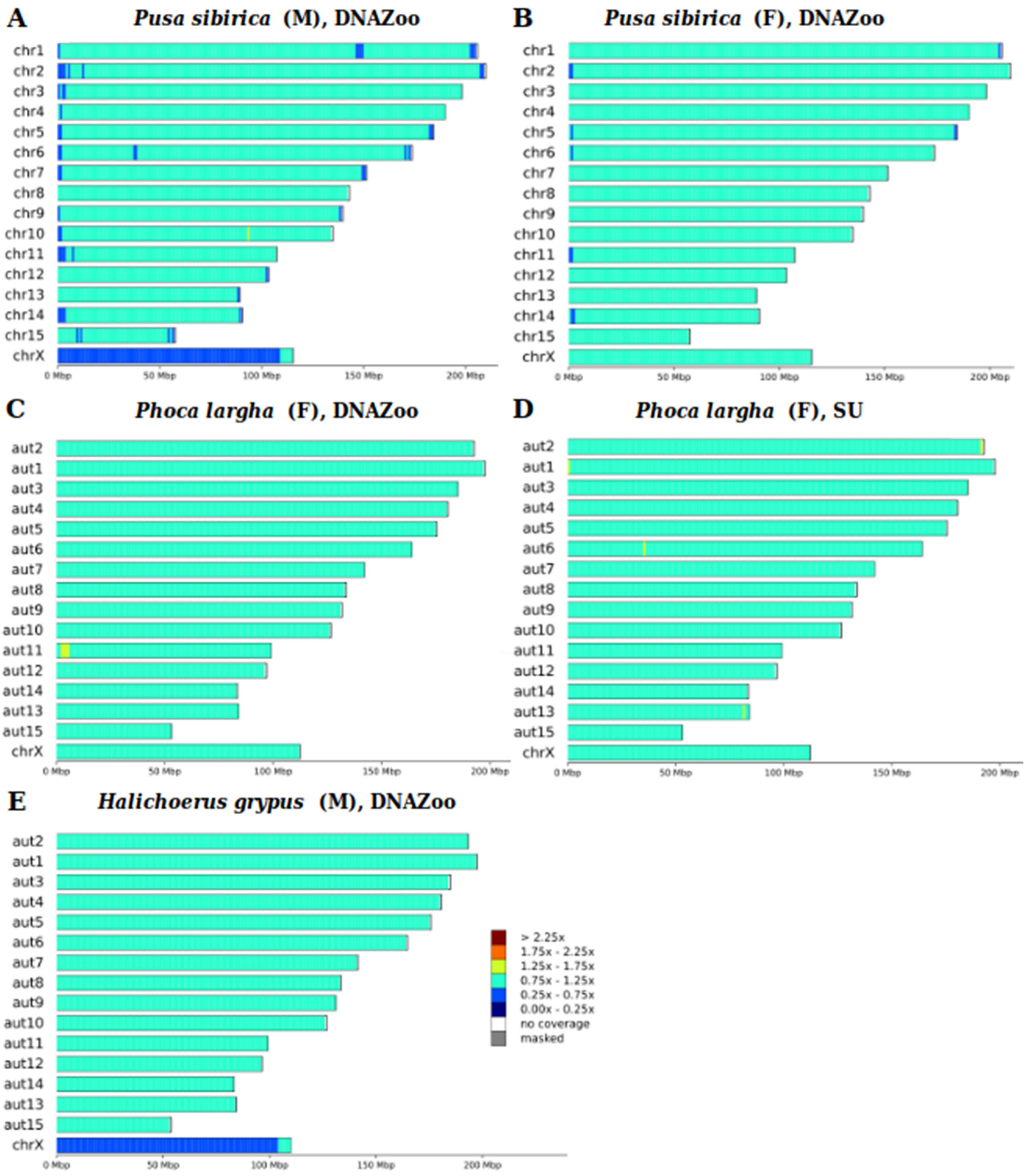

Figure S2. Coverage plots for (A) *Pusa sibirica,* female, DNAZoo, (B) *Pusa sibirica,* male, DNAZoo, (C) *Halichoerus grypus,* male, DNAZoo, (D) *Phoca largha,* female, DNAZoo, and (E) *Phoca largha,* female, Seoul University. Coverage was calculated in non-overlapping sliding windows of 1 Mbp and divided by whole genome median coverage.

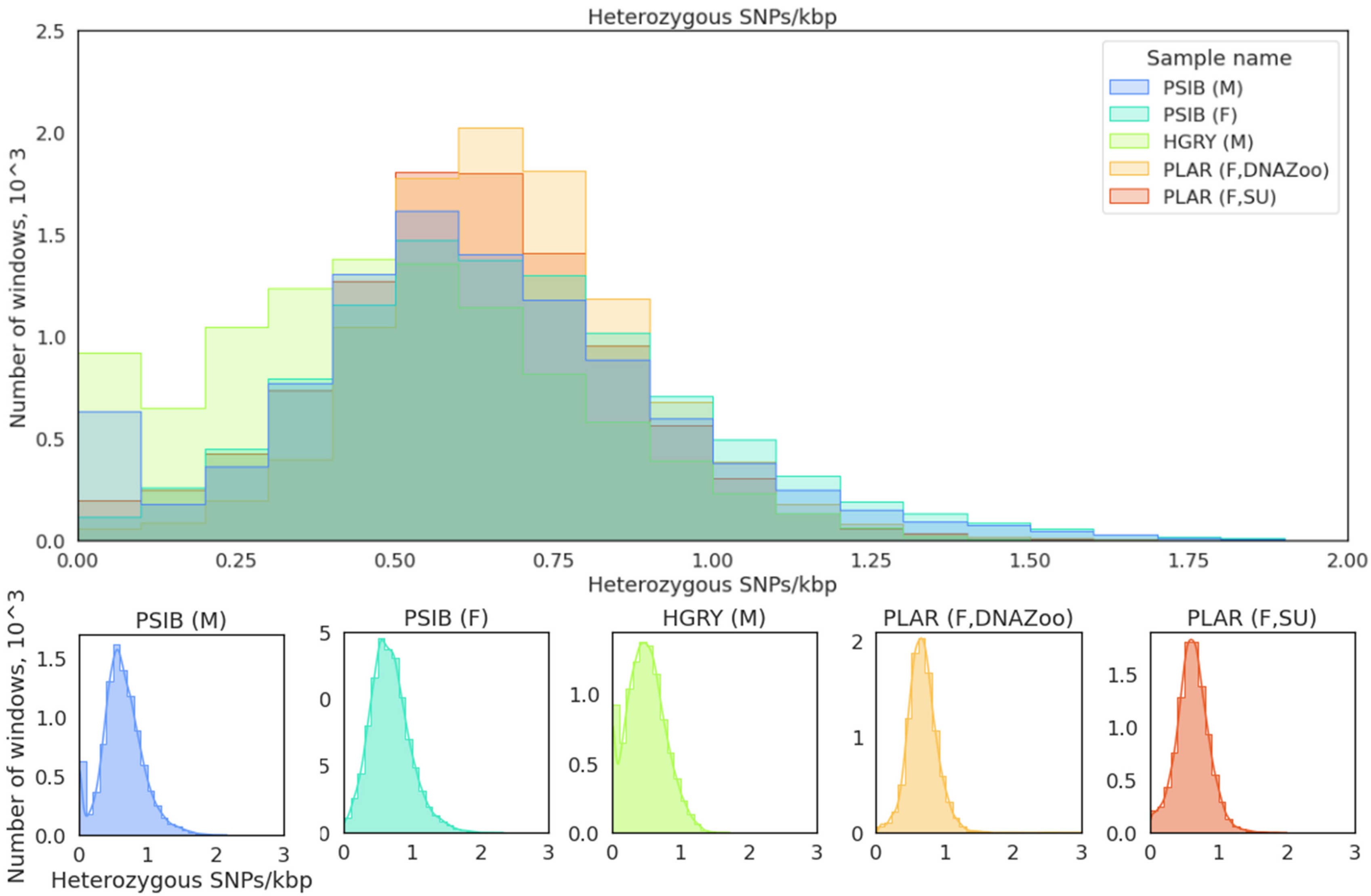

Figure S3. Genome-wide heterozygosity distribution for 2 samples of baikal seal (*Pusa sibirica*), 1 sample of grey seal (*Halichoerus grypus*) and 2 samples of spotted seal (*Phoca largha*). SNPs are counted in 1 Mbp non-overlapping sliding windows and scaled to heterozygous SNPs per kbp.

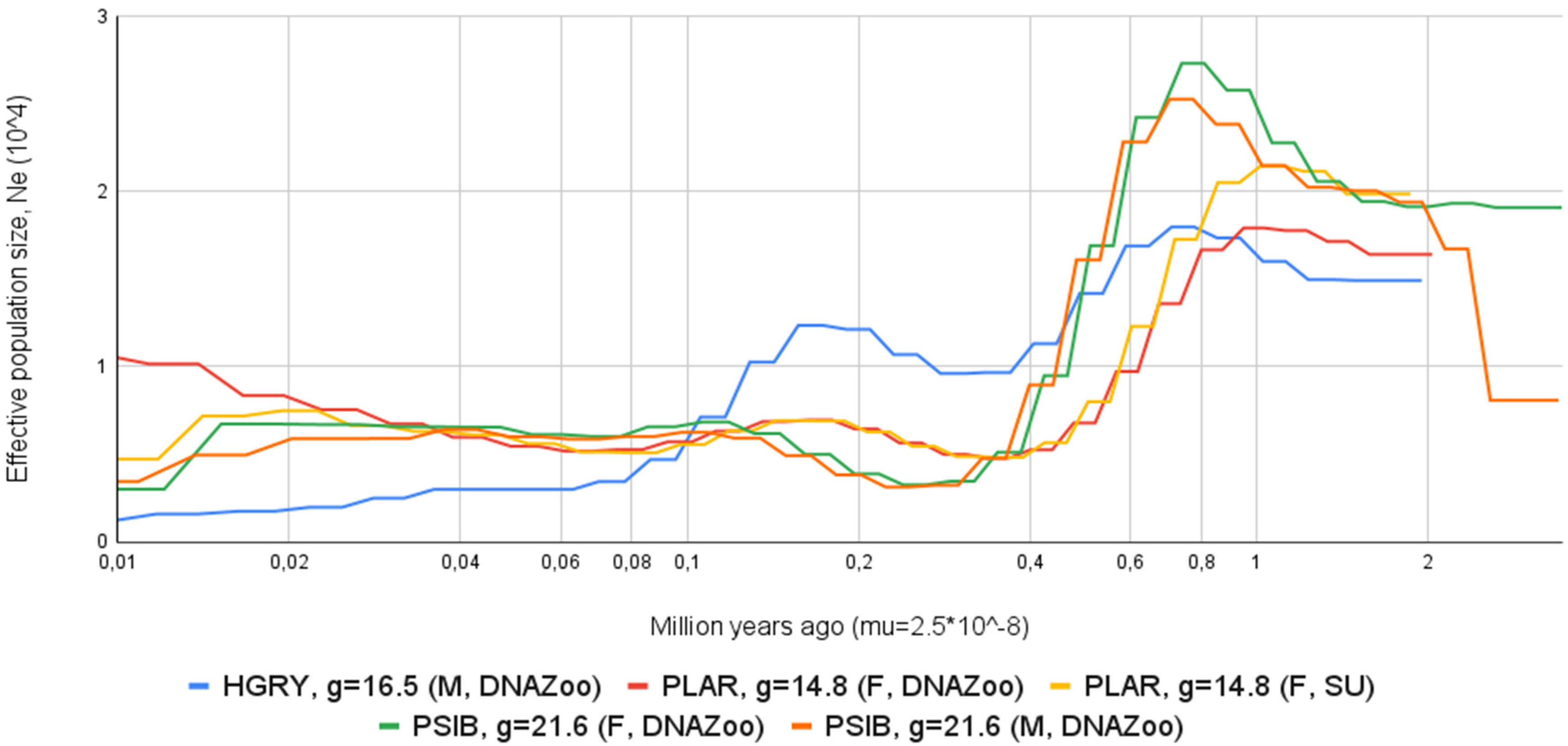

Figure S4. Demographic history reconstruction for all available samples of *Pusa sibirica, Halichoerus grypus*, and *Phoca largha* with mutation rate $2.5{\times}10^{-8}$ excluding X chromosome.

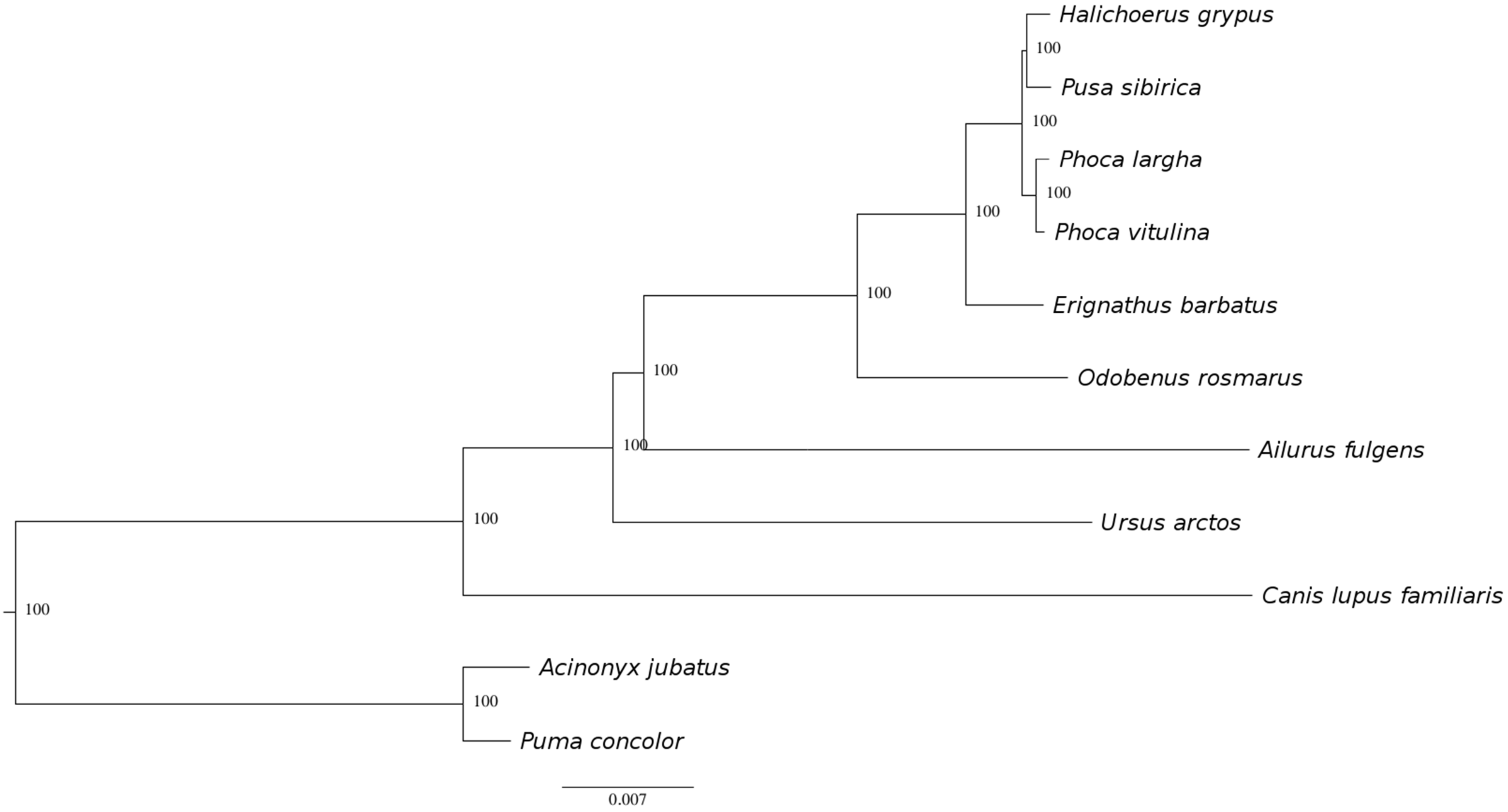

Figure S5. The phylogenetic tree for eleven carnivores including five seal species (*Halichoerus grypus, Pusa sibirica, Phoca largha, Phoca vitulina* and *Erignathus barbatus*). Node labels show posterior probabilities. Branch lengths are in proportion to expected changes per site.
